# Treatment-Seeking Behavior after the Implementation of a Unified Policy of Dihydroartemisinin-Piperaquine for the Treatment of Uncomplicated Malaria in Papua, Indonesia

**DOI:** 10.4269/ajtmh.17-0680

**Published:** 2017-12-26

**Authors:** Angela Devine, Enny Kenangalem, Faustina Helena Burdam, Nicholas M. Anstey, Jeanne Rini Poespoprodjo, Ric N. Price, Shunmay Yeung

**Affiliations:** 1 Mahidol-Oxford Tropical Medicine Research Unit, Mahidol University, Bangkok, Thailand;; 2 Centre for Tropical Medicine and Global Health, Nuffield Department of Clinical Medicine, University of Oxford, Oxford, United Kingdom;; 3 Timika Malaria Research Program, Papuan Health and Community Development Foundation, Timika, Papua, Indonesia;; 4 Mimika District Health Authority, Timika, Papua, Indonesia;; 5 Global and Tropical Health Division, Menzies School of Health Research and Charles Darwin University, Darwin, Northern Territory, Australia;; 6 Department of Child Health, Faculty of Medicine, University Gadjah Mada, Yogyakarta, Indonesia;; 7 Faculty of Infectious and Tropical Diseases, London School of Hygiene & Tropical Medicine, London, United Kingdom

## Abstract

Artemisinin combination therapy is recommended for the treatment of multidrug resistant *Plasmodium falciparum* and *Plasmodium vivax*. In March 2006, antimalarial policy in Indonesia was changed to a unified treatment with dihydroartemisinin-piperaquine for all species of malaria because of the low efficacy of previous drug treatments. In 2013, a randomized cross-sectional household survey in Papua was used to collect data on demographics, parasite positivity, treatment-seeking behavior, diagnosis and treatment of malaria, and household costs. Results were compared with a similar survey undertaken in 2005. A total of 800 households with 4,010 individuals were included in the 2013 survey. The prevalence of malaria parasitemia was 12% (348/2,795). Of the individuals who sought treatment of fever, 67% (66/98) reported attending a public provider at least once compared with 46% (349/764) before policy change (*P* < 0.001). During the 100 visits to healthcare providers, 95% (95) included a blood test for malaria and 74% (64/86) resulted in the recommended antimalarial for the diagnosed species, the corresponding figures before policy change were 48% (433/894) and 23% (78/336). The proportion of individuals seeking treatment more than once fell from 14% (107/764) before policy change to 2% (2/98) after policy change (*P* = 0.005). The mean indirect cost per fever episode requiring treatment seeking decreased from US$44.2 in 2005 to US$33.8 in 2013 (*P* = 0.006). The implementation of a highly effective antimalarial treatment was associated with better adherence of healthcare providers in both the public and private sectors and a reduction in clinical malaria and household costs.

## INTRODUCTION

In Indonesia, the greatest prevalence of malaria is in the eastern provinces of Nusa Tenggara Timur and Papua.^1^ Great progress has been made in reducing the burden of malaria with the majority (74%) of the Indonesian population residing in malaria-free areas. However, 12% of the population still reside in areas with a malaria incidence greater than one case per 1,000 per year,^2^ and these individuals are at increased risk of malaria associated anemia.^3,4^ Until 2006, the first line policy was chloroquine plus sulfadoxine-pyrimethamine and a single dose of primaquine for uncomplicated *Plasmodium falciparum* malaria, and chloroquine plus primaquine (total dose 3.5 mg/kg over 14 days) for radical cure for *Plasmodium vivax* with the exception of infants less than 1 year of age and pregnant or lactating women for whom chloroquine alone was recommended.^5^ In 2005, clinical trials in Papua, Indonesia, highlighted very high levels of antimalarial drug resistance to the recommended treatment regimens with recrudescent *P. falciparum* and recurrent *P. vivax* infections exceeding 40% by day 28 after treatment.^5–7^ Dihydroartemisinin-piperaquine (DHP) was shown to be highly effective against both species.^8,9^ In response, national policy was changed in March 2006 to the fixed dose artemisinin combination therapy (ACT) of DHP for uncomplicated malaria due to any species of malaria.^8,9^ The recommendation of single dose primaquine for *P. falciparum* remained, whereas the dose of primaquine recommended for *P. vivax* was increased to a total of 7 mg/kg over 14 days. The policy also stipulated that antimalarial treatment should only be given after laboratory confirmation, which was not a recommendation before 2006.

Before the change in policy, a cross-sectional household survey was undertaken in 2005, which documented that 42% of individuals reported having had a febrile illness in the preceding month, for which they sought treatment multiple times, often at different healthcare facilities.^10^ Only 46% of these individuals reported seeking treatment at a public provider, despite provision of treatment for free in the public sector. Most households reported a monthly income less than US$500, hence each individual’s fever episode resulted a cost of at least 11% of the household income.^10^ After policy change, the DHP was provided free of charge at public health facilities but was also available at a cost in the private sector.

Changes in antimalarial policy can potentially impact the overall burden and transmission of malaria not only through the direct impact of switching to a more effective treatment but also through increasing the community’s trust in the public health care system and the subsequent shifts in treatment-seeking behavior. The aim of this study was to describe the changes in treatment-seeking behavior for fever and burden of disease in Papua after policy change to ACT for all species of malaria.

## MATERIALS AND METHODS

Household surveys were conducted in Mimika Regency, southern Papua, Indonesia, in 2005 and 2013. The methods in 2013 were based on the 2005 survey which has been reported previously.^10^ Minor differences between the questionnaires included the collection of household income as a range rather than a specific estimate and information on bed net use by individuals rather than ownership by the household.

The demographics, malaria transmission, and health services in this region have been described previously.^3,4,11^ In brief, the district covers an area of 21,522 km^2^ with a population of approximately 120,457 in 2005 expanding to 202,350 in 2013. Most of the population resides in 12 districts, incorporating Timika town and the surrounding villages. The area is covered with thick rain forest with both coastal and mountainous areas, inhabited by a variety of ethnic groups which are broadly categorized as lowland Papuans, highland Papuans, or non-Papuans. Malaria transmission occurs throughout the year and is restricted to the lowland area where most of the population lives. There are three mosquito vectors: *Anopheles koliensis*, *Anopheles farauti*, and *Anopheles punctulatus*.

Healthcare provision is provided free of charge at public providers, which includes one hospital, 12 Government-funded Primary Health Clinics (Puskesmas), and 10 malaria control clinics provided by a local mining company. In 2008, a second hospital was opened, providing care to approximately 100,000 patients per year. Antimalarials can also be purchased in the private sector, including clinics, pharmacies, and drug stores.^12^ Diagnostic tests for malaria are available in the private sector but usually not in drug stores.

The household sampling strategy was the same for both surveys with 800 households sampled using a three-stage cluster sampling procedure to identify 32 clusters of 25 households.^10,13^ First, the four most populous subdistricts (Mimika Baru, Kuala Kencana, Iwaka, Kwamki Narama, and Wania) were chosen from the 12 subdistricts based on their accessibility by road from the main town of Timika and because they included the majority (80%) of the district’s population. The 2013 survey took place in similar geographic areas as the 2005 survey, but during the intervening years some of the subdistricts had been renamed. Second, the number of clusters in each subdistrict was apportioned according to their relative populations. The houses were then assigned numbers based on their geographical location, and clusters of 25 houses were identified.

The 2013 survey was conducted in the months of April through July, whereas the 2005 survey was conducted between the months of July and December. Household members were defined as all individuals living under one roof and who ate from one kitchen. If a selected household was empty, the interviewer returned the next day. If still unoccupied, the next occupied household was sampled instead. As the recall period for the survey was fever in the previous month, temporary migrants who had moved into the area within the preceding month were excluded from the survey.

### Ethics.

This study was approved by the Ethics Committee of the National Institutes of Health research and Development, Indonesian Ministry of Health and the Human Research (KS.02.01.2.1.2684), University of Gadjah Mada (KE/FK/763/EC), and the Ethics Committee of Northern Teritorry Department of Health and Community Services and Menzies School of Health Research, Darwin, Australia (HREC 04/47 and 10-1434). Written informed consent was obtained from all adult participants and parents of enrolled children.

### Data collection.

The head of the household or another suitable adult was asked questions about the household and its members. Those members who were present at the time of the survey underwent a brief examination. Capillary or venous blood samples were collected for blood film examination. Symptomatic illness was defined as a history of fever in the past 24 hours or an axillary temperature of 37.5°C or higher when examined. Nearly all surveys were conducted in Bahasa Indonesian and the remaining in a local Papuan language. Individuals reporting a history of fever in the preceding 30 days were asked to complete a separate module with detailed questions on treatment-seeking if they were present. This module comprised questions on all of the places where they had sought treatment, whether they had received a blood test, whether they were prescribed antimalarials or other medicines, and any associated costs to the households.

### Data analysis.

Data were analyzed using the STATA statistical software (version 14.2, StataCorp, College Station, TX)^14^ and R (version 3.2.3).^15^ A Discriminant Analysis of Principal Components was used to categorize households by socioeconomic status (SES) by data on reported ownership of assets.^10,16^ To ensure that sufficient time had elapsed to capture treatment-seeking behavior, those individuals reporting fever starting within the preceding 2 days were excluded from the analysis of treatment seeking. For the use of diagnostics, laboratory tests, and antimalarial prescriptions, each patient–provider interaction (“visit”) was analyzed for public and private healthcare providers.

Frequencies and percentages were used for the descriptive data. Percentages were compared using the χ^2^ test. Differences in outcome distributions were evaluated with the Mann–Whitney test. Continuous variables were compared using the Spearman’s rank for correlation. When less than five observations were seen in a category, categorical outcomes were compared using the Fisher’s Exact test. Simple logistic regression adjusted for clustering by households was used to calculate odds ratios (ORs) for comparisons within the 2013 survey. For the analysis of reported fever in the past month, significant risk factors (*P* < 0.05) in the univariate analysis were included in a multiple logistic regression model and significant interactions between age, ethnicity, and significant variables were reported. The 2005 and 2013 surveys were compared using adjusted odds ratios (AORs) for age (0–4, 5–14, 15+), gender, household SES, and ethnicity (non-Papuan, highland Papuan, and lowland Papuan). The models were adjusted for clustering by household.

Costs were gathered in Indonesian rupiah converted into United States Dollars (US$) using the average exchange rates^17,18^ and then revised to the 2014 equivalent using the consumer price index for Indonesia.^19^ The mean cost and standard deviation (SD) per fever episode are reported. Direct costs included transportation for the patient and care and treatment, which included consultations, diagnosis, medication, overnight stay, and any other payments. Indirect costs included the amount of time that usual activities were reduced for the individual and any companions, caretakers, or substitute laborers. This time was valued using the mean reported wage for all.

## RESULTS

### Household characteristics.

**Figure 1.**
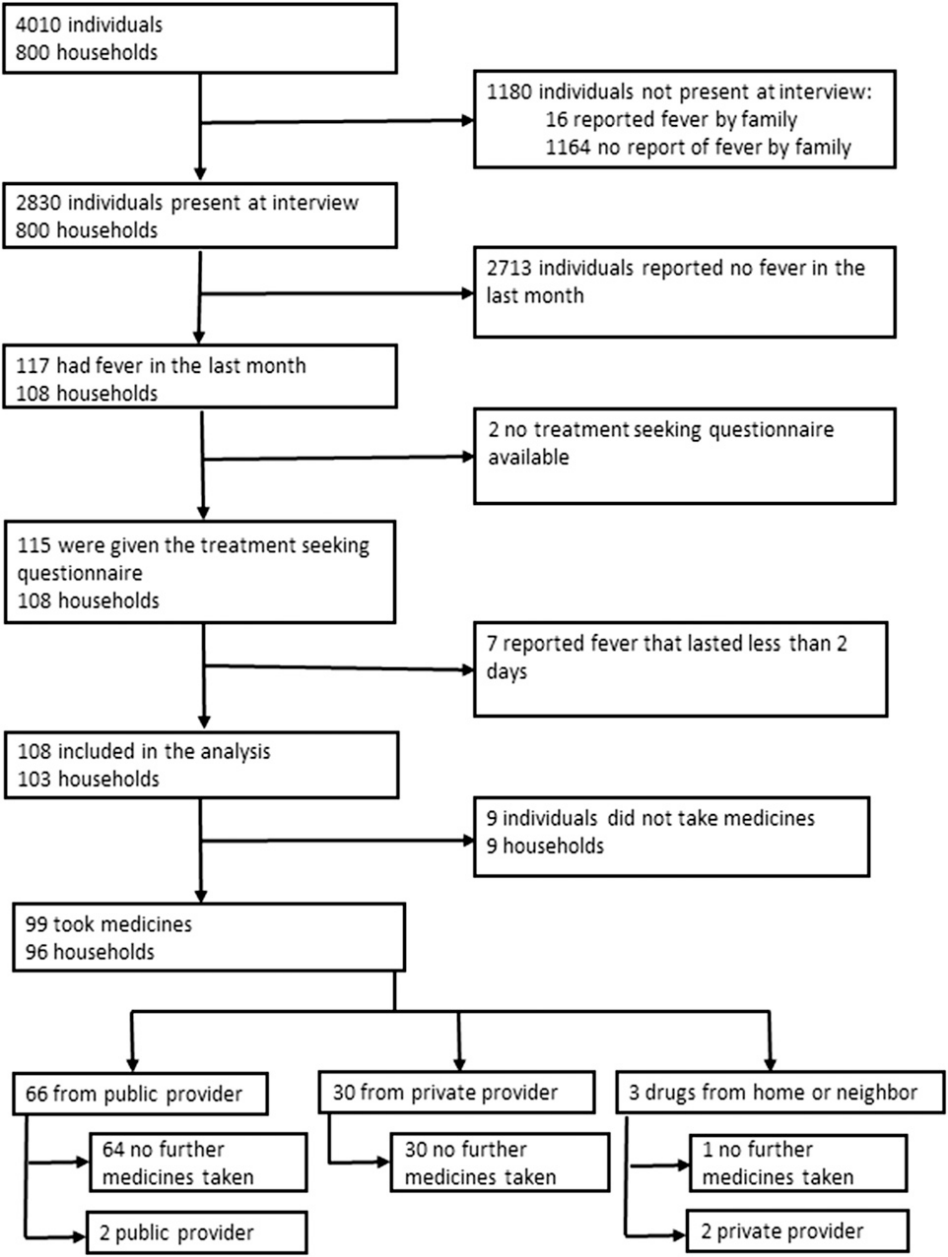
Flow diagram of household members, interviews, initial location of treatment taking, and whether they took a second treatment in 2013.

A total of 800 households with 4,010 individuals were included in the 2013 survey (Figure 1). The baseline characteristics for both the 2005 and 2013 surveys are provided in Table 1. In 2013, the median household size was five individuals (interquartile range [IQR] = 4–6) with a maximum of 13 in one house. The median duration that heads of households reported residing at their current location was 13 years (IQR = 6–20), with 23 (3%) households reporting a move into that location within the previous year. In 2005, the household size was six individuals (IQR = 4–8) with a median duration of 9 years (IQR = 4–15). Of the 781 households in which monthly household income was reported, 52% (403) estimated US$560 or less. The households were categorized into five SES groups, with the number of households in each group ranging from 91 to 296. While poorer households were more likely to grow their own crops and drink rain water, richer households were more likely to own motorcycles and large electrical items and drink bought water (Supplemental Figure 1). The poorest SES group was composed of a greater percentage of highland Papuans (32%, 47/145) compared with 19% (35/189) of lowland Papuans and 3% (15/465) of non-Papuans (*P* < 0.001). The estimated household general health expenditure was correlated with the number of individuals reporting fever in a household (ρ = 0.488, *P* < 0.001).

**Table 1:** Demographic characteristics of all household members and individuals present during surveys in 2005 and 2013

	2005		2013	
Characteristic, *n* (%)	All household members (*N* = 5,255)	Present during survey (*N* = 3,896)	All household members (*N* = 4,010)	Present during survey (*N* = 2,830)
Age (years)				
0–4	870 (17%)	820 (21%)	629 (16%)	559 (20%)
5–14	1,101 (21%)	824 (21%)	970 (24%)	666 (24%)
15+	3,284 (62%)	2,252 (58%)	2,404 (60%)	1,604 (57%)
Not reported	–	–	7 (0.2%)	1 (0.04%)
Sex (female)	2,409 (46%)	2,019 (52%)	2,035 (51%)	1,668 (59%)
Pregnant (yes)	92 (2%)	87 (2%)	–*	45/2,825 (2%)
Place of birth				
Highland Papuan	1,494 (28%)	1,045 (27%)	733 (18%)	539 (19%)
Lowland Papuan	1,371 (26%)	1,024 (26%)	1,039 (26%)	751 (27%)
Non-Papuan	2,390 (45%)	1,827 (47%)	2,238 (56%)	1,540 (54%)
Resided in lowlands for more than 1 year? (yes)	4,969 (95%)	3,674 (94%)	3,862 (96%)	2,711 (96%)
Subdistrict				
Banti	152 (3%)	119 (3%)	0 (0%)	0 (0%)
Harapan and Kwamki Lama	901 (17%)	576 (15%)	111 (3%)	80 (3%)
Inauga	521 (10%)	405 (10%)	383 (10%)	291 (10%)
Kamoro Jaya	354 (7%)	273 (7%)	369 (9%)	257 (9%)
Kadun Jaya, Kaugapu and Pigapu	371 (7%)	286 (7%)	267 (7%)	210 (7%)
Limau Asri, Iwaka, Mulia Kencana and Naena Muktipura	136 (3%)	112 (3%)	506 (13%)	334 (12%)
Koperapoka	957 (18%)	729 (19%)	753 (19%)	521 (18%)
Timika Jaya, Kwamki and Kwamki Baru	1,407 (27%)	1,046 (27%)	989 (25%)	696 (25%)
Wonosari Jaya and Nawaripi	122 (2%)	98 (3%)	374 (9%)	267 (9%)
Karang Senang and Bhintuka	334 (6%)	252 (6%)	258 (6%)	174 (6%)

* Not reported.

### History of fever and parasite positivity of household members.

Of the 2,830 (71%) individuals present at the time of the interview in 2013, 41% (1,162) were male, 43% (1,225) were under the age of 15, and 46% (1,290) were Papuan (Table 1). Overall, 4% (117/2,830) of individuals reported having had a febrile illness in the preceding month compared with 42% (1,631/3,896) in 2005 (AOR = 0.06, 95% confidence interval [CI] = 0.05–0.08, *P* < 0.001; Table 2). Non-Papuan participants were at a significantly higher risk for having had a febrile illness compared with highland Papuans (AOR = 1.84, 95% CI = 1.05–3.26, *P* = 0.035; Supplemental Table 1).

**Table 2:** Fever reporting in those who were present during the survey in 2005 and 2013

	2005		2013	
Characteristic, *n* (%)	Reported fever during previous month (*N* = 1,631/3,896, 42%)	Reported fever starting > 2 days before survey and took treatment (*N* = 834/1,631, 51%)	Reported fever during previous month (*N* = 117/2,830, 4%)	Reported fever starting > 2 days before survey and took treatment (*N* = 99/117, 85%)
Age (years)				
0–4	405 (25%)	205 (25%)	23 (20%)	15 (15%)
5–14	306 (19%)	148 (18%)	29 (25%)	26 (26%)
15+	920 (56%)	481 (58%)	65 (56%)	58 (59%)
Sex (female)	850 (52%)	433 (52%)	63 (54%)	55 (56%)
Pregnant (yes)	39 (2%)	23 (3%)	3/116 (3%)	3/98 (3%)
Place of birth				
Highland Papuan	385 (24%)	172 (21%)	14 (12%)	11 (11%)
Lowland Papuan	463 (28%)	183 (22%)	28 (24%)	19 (19%)
Non-Papuan	783 (48%)	479 (57%)	75 (64%)	69 (70%)
Resided in lowlands for more than 1 year? (yes)	1,547 (95%)	797 (96%)	111 (95%)	93 (94%)
Subdistrict				
Banti	29 (2%)	18 (2%)	0 (0%)	0 (0%)
Harapan and Kwamki Lama	226 (14%)	124 (15%)	4 (3%)	2 (2%)
Inauga	183 (11%)	106 (13%)	5 (4%)	5 (5%)
Kamoro Jaya	107 (7%)	77 (9%)	12 (10%)	10 (10%)
Kadun Jaya, Kaugapu and Pigapu	108 (7%)	11 (1%)	23 (20%)	14 (14%)
Limau Asri, Iwaka, Mulia Kencana and Naena Muktipura	55 (3%)	3 (0.4%)	15 (13%)	15 (15%)
Koperapoka	396 (24%)	127 (15%)	13 (11%)	12 (12%)
Timika Jaya, Kwamki and Kwamki Baru	383 (23%)	262 (31%)	16 (14%)	14 (14%)
Wonosari Jaya and Nawaripi	49 (3%)	39 (5%)	24 (21%)	23 (23%)
Karang Senang and Bhintuka	95 (6%)	67 (8%)	5 (4%)	4 (4%)

Overall, 12% (348/2,795) of individuals who consented to blood testing in 2013 were parasitemic, a slight reduction from 16% (634/3,890) in 2005 (AOR = 0.83, 95% CI = 0.69–0.99, *P* = 0.038). The change in prevalence was most apparent in individuals with symptomatic parasitemia which decreased from 5% (200/3,890) in 2005 to 1% (21/2,795) in 2013 (AOR = 0.16, 95% CI = 0.10–0.26, *P* < 0.001), whereas the prevalence of asymptomatic parasitemia was similar in both surveys: 11% (434/3,890) in 2005 and 12% (327/2,795) in 2013 (AOR = 1.20, 95% CI = 0.98–1.45, *P* = 0.073). Individuals reporting a febrile illness in the past month were significantly more likely to be parasitemic in the 2005 survey (OR = 3.81, 95% CI = 3.10–4.68, *P* < 0.001); however, this was not significant in the 2013 survey (OR = 1.53, 95% CI = 0.89–2.61, *P* = 0.123). The prevalence of parasitemia in each subdistrict is presented in Supplemental Table 2.

### Treatment-seeking behavior.

In 2013, treatment-seeking behavior was assessed in 98% (115/117) of participants reporting a fever in the preceding month, and in 94% (108/115) of these individuals the fever commenced at least 2 days before the survey (Figure 1). Individuals were more likely to take some form of medicine for their fever in 2013 with 92% (99/108) reporting taking medication compared with 76% (834/1,104) of those whose fever commenced at least 2 days before the survey in 2005 (AOR = 2.50, 95% CI = 1.23–5.06, *P* = 0.011). Of the 99 patients who reported taking a medication, only 1% (1) did not seek treatment outside of the home, compared with 8% (70/834) in 2005 (*P* = 0.004). In 2013, 67% (66/98) of individuals seeking treatment outside of the home attended a public provider at least once compared with 46% (349/764) in 2005 (AOR = 4.30, 95% CI = 2.54–7.28, *P* < 0.001). Furthermore, individuals in the three lower SES groups were more likely to seek treatment at a public provider at least once compared with those in the top two SES groups (OR = 6.10, 95% CI = 2.05–18.11, *P* = 0.001); this was also apparent in 2005 (OR = 2.29, 95% CI 1.59–3.32, *P* < 0.001).

### Diagnosis of malaria at healthcare providers.

Of the 100 visits to healthcare providers in 2013, 95 (95%) involved a blood test for malaria compared with 48% (433/894) in 2005 (AOR = 24.42, 95% CI = 9.87–65.43, *P* < 0.001); Table 3. Malaria testing was positive in 92% (87/95) of cases and in one visit the result was not reported. *Plasmodium falciparum* was diagnosed in 53% (46/87) of cases, *P. vivax* in 43% (37/87), mixed infections in 3% (3/87), and *Plasmodium malariae* in 1% (1/87). The increase in likelihood of individuals being tested for malaria was most apparent in the private sector where 29% (148/519) of individuals were tested in 2005 compared with 97% (31/32) in 2013 (*P* < 0.001).

**Table 3:** Percentage of respondents who reported receiving blood tests and antimalarials (including tablets and injections) by survey

	Public				Private				Overall			
	2005	2013	AOR (95% CI)*	*P* value	2005	2013	AOR (95% CI)*	*P* value	2005	2013	AOR (95% CI)*	*P* value
Percent of visits that resulted in a blood test for malaria	76% (285/375)	94% (64/68)	–	< 0.001	29% (148/519)	97% (31/32)	–	< 0.001	48% (433/894)	95% (95/100)	25.42 (9.88–65.42)	< 0.001
Percent of blood tests that were positive	75% (213/285)	94% (60/64)	–	< 0.001	84% (124/148)	87% (27/31)	–	0.790	78% (337/433)	92% (87/95)	3.11 (1.29–7.47)	0.012
Percent receiving correct antimalarial after testing positive†	32% (67/212)	85% (50/59)	15.94 (6.84–37.14)	< 0.001	9% (11/124)	52% (14/27)	12.16 (3.42–43.28)	< 0.001	23% (78/336)	74% (64/86)	15.71 (8.18–30.18)	< 0.001
Percent receiving chloroquine after testing positive	62% (133/212)	0% (0/59)	–	< 0.001	43% (53/124)	11% (3/27)	–	0.002	55% (186/336)	3% (3/86)	–	< 0.001
Percent not receiving an antimalarial after testing negative	98% (61/62)	100% (3/3)	–	–‡	86% (19/22)	100% (4/4)	–	–‡	95% (80/84)	100% (7/7)	–	–‡
Percent not receiving an antimalarial if no blood test given	64% (58/90)	100% (4/4)	–	–‡	52% (192/371)	0% (0/1)	–	–‡	54% (250/461)	80% (4/5)	–	0.383

AOR = adjusted odds ratio; CI = confidence interval.

* Multivariate logistic regression adjusted for age, sex, ethnicity, and household socioeconomic status if five or more observations. The Fisher’s exact if less than five observations (no AORs).

† In 2005, local guidelines recommended antimalarial treatment with chloroquine plus sulphadoxine–pyramethamine for *Plasmodium falciparum* and chloroquine for *Plasmodium vivax*. In those patients with *P. vivax* (either alone or mixed) primaquine was also recommended. In 2013, correct treatment regimens were dihydoartemisinin-piperaquine for all species of malaria plus primaquine in those with *P. vivax* infections (alone or mixed). *Plasmodium malariae* infections (*N* = 2) were excluded from this analysis.

‡ 2013 data predicted the outcome perfectly. All individuals had the same level of outcome; hence, no statistical computations can be performed for the logistic regression—no statistical output is given in software.

### Antimalarial treatment at healthcare providers.

In 2013, an antimalarial (alone or with other medicines) was reportedly prescribed during 88% (88/100) of visits to healthcare providers. Most of these prescriptions were given as tablets (95%, 84/88) with one (1%) injection and three (3%) for both an injection and tablets. Of the four malaria injections that were reported, two were at the public hospital and two at private providers. Ninety-nine percent (87/88) of antimalarial prescriptions followed a blood test as per clinical guidelines; all were reportedly positive. This was a significant increase compared with 2005 when only 61% (336/547) of prescriptions followed a blood test (*P* < 0.001). In 2013, the antimalarial treatment regimen was prescribed correctly in 74% (64/86) of healthcare encounters in which *P. vivax*, *P. falciparum*, or mixed infections were diagnosed, a significant increase from 2005 when only 23% (78/336) of individuals were correctly treated according to the prevailing guidelines (AOR = 15.71, 95% CI = 8.18–30.18, *P* < 0.001; Table 3). These improvements in prescribing habits were apparent in both the public (AOR = 15.93, 95% CI = 6.84–37.14, *P* < 0.001) and private sectors (AOR = 12.16, 95% CI = 3.42–43.28, *P* < 0.001).

The reported treatments prescribed for the different species are presented in Table 4. In 2013, 95% (57/60) of patients diagnosed with malaria in a public facility were treated with DHP compared with 52% (14/27) of those treated at a private provider (*P* < 0.001). Between 2005 and 2013, the percentage of individuals reportedly being treated with 14 days of primaquine for *P. vivax* infection (alone or mixed) rose from 9% (16/169) to 63% (25/40); (AOR = 37.49, 95% CI = 9.94–141.41, *P* < 0.001). Over the same period, the percentage of patients with *P. falciparum* reporting treatment with a single dose primaquine rose from 19% (31/167) in 2005 to 50% (23/46) in 2013 (AOR = 6.27, 95% CI = 2.68–14.68, *P* < 0.001).

**Table 4:** Reported antimalarial treatment of individuals by reported diagnosis

	2005 (*N* = 336)			2013 (*N* = 86)		
	Private	Public	Overall	Private	Public	Overall
*Plasmodium falciparum* (*N*)	68	99	167	14	32	46
Percentage receiving any ACT	0% (0)	1% (1)	1% (1)	50% (7)	97% (31)	83% (38)
Percentage receiving any antimalarial without ACT	88% (60)	94% (93)	92% (153)	36% (5)	3% (1)	13% (6)
Percentage receiving unknown antimalarial	0% (0)	3% (3)	2% (3)	14% (2)	0% (0)	4% (2)
Percentage receiving primaquine (any dose)	62% (42)	82% (81)	74% (123)	71% (10)	59% (19)	63% (29)
Percentage receiving any treatment	94% (64)	98% (97)	96% (161)	100% (14)	100% (32)	100% (46)
Percentage receiving no treatment	6% (4)	2% (2)	4% (6)	0% (0)	0% (0)	0% (0)
*Plasmodium vivax* (*N*)	51	108	159	12	25	37
Percentage receiving any ACT	0% (0)	0% (0)	0% (0)	50% (6)	96% (24)	81% (30)
Percentage receiving any antimalarial without ACT	75% (38)	94% (102)	88% (140)	25% (3)	0% (0)	8% (3)
Percentage receiving unknown antimalarial	20% (10)	5% (5)	9% (15)	25% (3)	4% (1)	11% (4)
Percentage receiving primaquine (any dose)	29% (15)	81% (88)	65% (103)	58% (7)	72% (18)	68% (25)
Percentage receiving any treatment	94% (48)	99% (107)	97% (155)	100% (12)	100% (25)	100% (37)
Percentage receiving no treatment	6% (3)	1% (1)	3% (4)	0% (0)	0% (0)	0% (0)
Mixed infections (*N*)	5	5	10	1	2	3
Percentage receiving any ACT	0% (0)	0% (0)	0% (0)	100% (1)	50% (1)	67% (2)
Percentage receiving any antimalarial without ACT	80% (4)	60% (3)	70% (7)	0% (0)	0% (0)	0% (0)
Percentage receiving unknown antimalarial	0% (0)	40% (2)	20% (2)	0% (0)	50% (1)	33% (1)
Percentage receiving primaquine (any dose)	60% (3)	60% (3)	60% (6)	100% (1)	50% (1)	67% (2)
Percentage receiving any treatment	80% (4)	100% (5)	90% (9)	100% (1)	100% (2)	100% (3)
Percentage receiving no treatment	20% (1)	0% (0)	10% (1)	0% (0)	0% (0)	0% (0)

ACT = antimalarial combination therapy.

### Cost burden of fever.

In 2013, a total of 100 visits to healthcare providers were reported by 98 individuals; hence, nearly all patients reported requiring only one visit compared with 14% (107/764) of individuals who reported seeking medical care at two or more providers before the policy change (*P* < 0.001). In 2013, the mean total direct cost to the household per visit was US$16.8 (SD = 19.0) at a private provider and US$4.1 (SD = 14.4) at the public sector (*P* < 0.001). The mean daily wage in 2013 was US$18.2 (SD = 12.5), with a total mean indirect cost per fever episode of US$52.5 (SD = 41.4) for adults and US$7.3 (SD = 16.4) for children.

Table 5 shows the cost per fever episode resulting in treatment seeking in 2005 and 2013. While the direct costs were similar (*P* = 0.119), a significant decrease in indirect costs was evident from US$44.2 (SD = 51.0) before the policy change to US$33.8 (SD = 40.1) after the policy change (*P* = 0.006). In 2013, only 29% (29/99) of individuals reported that someone had had to cut back on their usual activities to care for them, a decrease from 77% (641/834) in 2005 (OR = 0.12, 95% CI = 0.08–0.20, *P* < 0.001). There was also a reduction in the number of individuals requiring a substitute laborer from 8% (66/834) in 2005 to 1.0% (1/99) in 2013 (*P* = 0.007). There was no significant difference in the proportion of individuals having at least one companion accompany them to seek treatment (66% (553/834) in 2005 versus 73% (72/99) in 2013; *P* = 0.200). Overall, the mean cost per fever episode was US$41.9 (SD = 44.3) in 2013 compared with US$53.5 (SD = 65.3) in 2005 (*P* = 0.104).

**Table 5:** Reported direct, indirect, and total costs per individual taking treatment of fever over the entire fever episode in 2014 United States dollars

Category	2005 (*N* = 834)		2013 (*N* = 99)		*P* value*
	Mean (SD)	Median (IQR)	Mean (SD)	Median (IQR)	
Total direct costs†	9.4 (27.3)	1.9 (0.4–5.6)	8.1 (17.0)	1.7 (1.0–8.1)	0.119
Total indirect costs	44.2 (51.0)	32.8 (10.9–60.1)	33.8 (40.1)	18.2 (0.0–54.6)	0.006
Total cost to the individual taking treatment due to lost wages	19.3 (30.6)	0.0 (0.0–32.8)	28.4 (38.3)	9.1 (0.0–45.5)	0.010
Total cost for companions	4.1 (6.5)	0.00 (0.0–5.5)	3.2 (7.6)	0.0 (0.0–0.0)	0.001
Total cost for caretaking	19.7 (30.5)	10.9 (0.0–27.3)	2.0 (9.1)	0.0 (0.0–0.0)	< 0.001
Total cost for substitute labor	1.1 (6.5)	0.0 (0.0–0.0)	0.2 (1.8)	0.0 (0.0–0.0)	0.072
Total costs	53.5 (65.3)	33.8 (16.4–66.7)	41.9 (44.3)	27.3 (8.1–64.7)	0.104

IQR = interquartile range; SD = standard deviation.

* Mann–Whitney rank test.

† Includes payments made by the household for all treatments including diagnostics and transport.

## DISCUSSION

The results of the household surveys undertaken before and after the implementation of ACT after confirmed diagnosis in Papua, Indonesia, highlight several important changes in treatment-seeking behavior, diagnosis of malaria, and adherence to antimalarial drugs. Only one individual (1%) did not seek treatment outside of the home compared with 8% in 2005 when less efficacious treatments were available in both the public and private sectors. This shift in treatment seeking was associated with more individuals attending public providers at least once, rising from 46% in 2005 to 67% in 2013. Those from households with lower SES were still more likely to seek treatment at public providers than those with higher SES.

Before the implementation of ACT, more than 40% of patients failed antimalarial treatment within 42 days, with almost half of these patients doing so within 7 days.^5^ The introduction of a 3-day regimen of DHP resulted in rapid parasite clearance and symptom recovery with less than 5% of patients having recurrent malaria, most of which occurred after 28 days.^8^ Hence, the observed changes in treatment-seeking behavior likely reflect community awareness of such highly effective treatments and their availability initially in the public sector and subsequently in the private sector.

In parallel with the shift in where patients sought treatment, there were also significant changes in the way in which healthcare services were provided, particularly the treatment of malaria only after laboratory confirmation and adherence to antimalarial policy. Rapid diagnostic tests were introduced at public health facilities without microscopists in 2006–2007 and were used for half of the outpatient visits to one of the hospitals, whereas microscopy remained the main diagnostic for patients requiring admission to hospital. In 2013, individuals who attended healthcare facilities were almost twice as likely to have a blood test for malaria compared with those in 2005, and this was particularly prominent in the private sector where almost all patients were offered a blood test in 2013. Considering that dissatisfaction with the public sector is common^20^ and studies in Southeast Asia have shown that a large proportion of individuals seek treatment in the private sector,^21,22^ improvement of both sectors is vital to ensuring public health benefits.^23^

Our findings also demonstrate that individuals diagnosed with malaria in 2013 were 3-fold more likely to receive the recommended treatment regimen. Importantly, both the public and private sectors implemented the policy change to ACT, with reductions in prescriptions of non-ACT antimalarials apparent in both sectors. This included an 8-fold decline in chloroquine prescriptions. Furthermore, better prescribing practices were also observed for primaquine, which is encouraged as a single dose for reducing the transmission potential of *P. falciparum* and as a 14-day regimen for the radical cure of *P. vivax*.

The survey results also highlight a modest fall in the prevalence of parasitemia from 16% to 12%, although individuals who were parasitemic were 6-fold less likely to be symptomatic. Furthermore, a 10-fold reduction (from 42% to 4%) was observed in those reporting fever in the preceding month. Before policy change, individuals reporting a recent fever were three times more likely to be parasitemic at the time of the survey than those with no recent febrile illness. Since the risk an infected mosquito bite within 28 days in this region is low (< 8%), the most likely explanation for this is that before 2006 treatment with partially effective treatment often failed to clear parasitemia by the time of the survey, whereas this was no longer apparent after the change in policy to highly effective treatment with ACT. By contrast, no reduction was observed in the prevalence of asymptomatic parasitemia between 2005 and 2013, a likely reflection of these patients not being unwell enough to seek treatment.^24^

The first survey was conducted between July and December, whereas the second was undertaken from April through July. While seasonal variation in either malaria or other causes of fever could therefore have influenced our observations, malaria transmission in this area occurs throughout the year with minimal seasonality.^24^ Other factors that could have affected our results include fluctuations in the demography. Between 2005 and 2013, the population grew from 120,000 to 172,000, and this was complemented with a slight increase in the proportion of non-Papuans, who are generally less immune. But this was countered by individuals reporting having lived in the area longer which would facilitate greater asymptomatic infections. In 2013, the only significant risk factor for recent febrile illness was being non-Papuan (Supplemental Table 1). Whereas in 2005 lowland Papuans, children less than 5 years old, and those from lower SES household were all at greater risk.^10^ The changes in treatment-seeking behavior therefore likely reflect a complex shift in population dynamics coupled with the impact of better treatment regimens and healthcare provision.

The observed changes in treatment seeking had important implications for the household costs incurred by malaria. While the mean cost for each febrile episode decreased from US$53.5 to US$41.9, reported monthly income in households increased from 60% with less than US$500 in 2005 to 49% with less than US$560 in 2013. Furthermore, in 2005, households more frequently reported multiple household members with fevers in the past month and those household members reported repeat visits as compared with 2013. Considering the decrease in symptomatic individuals who were parasite positive, it is likely that many of these changes in treatment-seeking behavior are a result of effective treatment.

This study has a number of limitations. First, a degree of recall bias is likely due to the time between the fever episode and the survey, although this bias would be expected to be similar for both surveys. The 8-year gap between surveys increases confounding from other changes besides the policy change including demographic dynamics, such as smaller households, greater representation of females, and duration residing in the study area. Public health and malaria control activities aimed at changing behavior remained similar over this time period. Because of differences in the phrasing of the questionnaires, we were unable to explore how the use of bed nets had changed over time. As with the previous survey, many older male household members were not present at the time of the interviews and temporary migrants were excluded.

In conclusion, the policy change in March 2006 to a unified treatment of malaria with DHP has had a significant impact on treatment-seeking behavior, resulting in more patients attending healthcare facilities where better case management was being provided. These changes have had a significant impact on the burden of malaria with less patients reporting febrile illness; however, increases were seen in asymptomatic infections. Further reduction in malaria and its ultimate elimination will require implementing more effective radical cure of *P. vivax* and the implementation of active case detection or presumptive interventions.

## Supplementary Material

Supplemental Table and Figure.
