# Supplementary material for: Treatment-Seeking Behavior after the Implementation of a Unified Policy of Dihydroartemisinin-Piperaquine for the Treatment of Uncomplicated Malaria in Papua, Indonesia
Source: Am J Trop Med Hyg. 2017 Dec 26;98(2):543–50. doi: 10.4269/ajtmh.17-0680 (PMC5810904; doi:10.4269/ajtmh.17-0680)
Supplement: Supplementary file 1 [file tpmd170680.SD1.pdf]

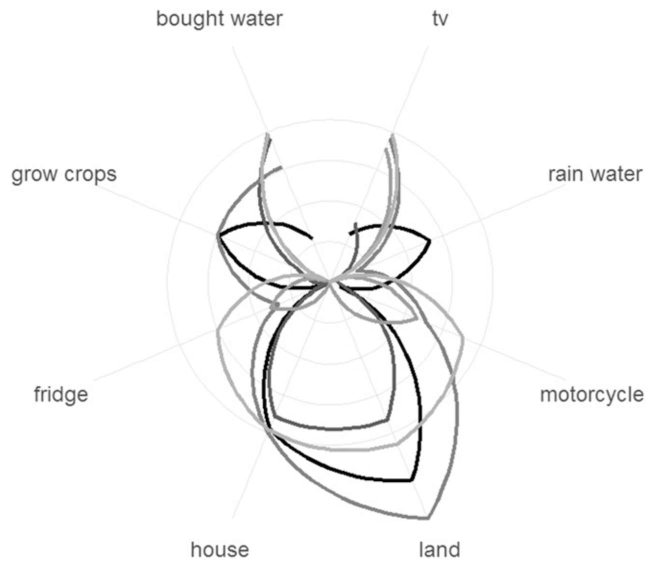

SUPPLEMENTAL FIGURE 1. Tree plot of ownership by groups from poorest (black) to richest (lightest gray). A discriminant analysis of principal components used to construct these groupings.

SUPPLEMENTAL TABLE 1
Multivariable analysis of risk factors for febrile illness in the past month (N = 2,830)

Variable	OR (95% CI)	*P* value	AOR (95% CI)	*P* value
Gender				
Female	Reference	–	–	–
Male	1.24 (0.85–1.82)	0.266	–	–
Age (years)				
> 15	Reference	–	–	–
5–14	1.08 (0.68–1.70)	0.746	–	–
< 5	1.02 (0.61–1.69)	0.951	–	–
Pregnant				
No	Reference	–	–	–
Yes	1.69 (0.51–5.55)	0.391	–	–
Household size				
> 7 members	Reference	–	Reference	–
≤ 7 members	2.11 (1.01–4.43)	0.048	2.00 (0.96–4.15)	0.062
Household socioeconomic status				
Richest	Reference	–	–	–
Fourth	0.68 (0.37–1.24)	0.211	–	–
Middle	1.32 (0.78–2.23)	0.302	–	–
Second	1.05 (0.61–1.80)	0.857	–	–
Poorest	0.76 (0.39–1.47)	0.410	–	–
Ethnicity				
Highland Papuan	Reference	–	Reference	–
Lowland Papuan	1.45 (0.74–2.84)	0.275	1.53 (0.79–2.97)	0.209
Non-Papuan	1.92 (1.08–3.40)	0.025	1.84 (1.05–3.26)	0.035
Resided in lowlands > 1 year?				
Yes	Reference	–	–	–
No	1.24 (0.54–2.84)	0.605	–	–
Individual sleeps under an insecticide-treated bednet?				
No	Reference	–	–	–
Yes	1.27 (0.86–1.86)	0.226	–	–

AOR = adjusted odds ratio; CI = confidence interval; OR = odds ratio. The multiple logistic regression included variables that were significant ($P < 0.05$) in the univariate logistic regression.

Supplemental Table 2

Changes in parasite prevalence by location (subdistrict)

Subdistrict	2005						2013					
			Species, *n* (%)						Species, *n* (%)			
	Household members	Total parasitemic *N*	*Plasmodium falciparum*	*Plasmodiuma vivax*	*Plasmodium malariae*	Mixed infection	Household members	Total parasitemic *N*	*Plasmodium falciparum*	*Plasmodium vivax*	*Plasmodium malariae*	Mixed infection
Banti	119	7 (6%)	0 (0%)	7 (100%)	0 (0%)	0 (0%)	0	–	–	–	–	–
Harapan and Kwamki Lama	576	136 (24%)	67 (49%)	56 (41%)	2 (2%)	11 (8%)	80	14 (18%)	6 (43%)	4 (29%)	4 (29%)	0 (0%)
Inauga	405	47 (12%)	19 (40%)	23 (49%)	0 (0%)	5 (11%)	290	25 (9%)	10 (40%)	14 (56%)	1 (4%)	0 (0%)
Kamoro Jaya	273	102 (37%)	44 (43%)	24 (24%)	11 (11%)	23 (23%)	254	37 (15%)	17 (46%)	14 (38%)	5 (14%)	1 (3%)
Kadun Jaya, Kaugapu, and Pigapu	285	35 (12%)	10 (29%)	17 (49%)	2 (6%)	6 (17%)	198	60 (30%)	29 (48%)	25 (42%)	1 (2%)	5 (8%)
Limau Asri, Iwaka, Mulia Kencana, and Naena Muktipura	111	5 (5%)	3 (60%)	2 (40%)	0 (0%)	0 (0%)	330	27 (8%)	17 (63%)	9 (33%)	1 (4%)	0 (0%)
Koperapoka	726	100 (14%)	42 (42%)	45 (45%)	1 (1%)	12 (12%)	517	58 (11%)	27 (47%)	31 (53%)	0 (0%)	0 (0%)
Timika Jaya, Kwamki, and Kwamki Baru	1,045	133 (13%)	68 (51%)	57 (43%)	1 (0.8%)	7 (5%)	695	60 (9%)	17 (28%)	40 (67%)	2 (3%)	1 (2%)
Wonosari Jaya and Nawaripi	98	15 (15%)	11 (73%)	4 (27%)	0 (0%)	0 (0%)	258	37 (14%)	14 (38%)	16 (43%)	6 (16%)	1 (3%)
Karang Senang and Bhintuka	252	54 (21%)	26 (48%)	13 (24%)	7 (13%)	8 (15%)	173	30 (17%)	12 (40%)	10 (33%)	5 (17%)	3 (10%)
Total	3,890	634 (16%)	290 (46%)	248 (39%)	24 (4%)	72 (11%)	2,795	348 (12%)	149 (43%)	163 (47%)	25 (7%)	11 (3%)
